# MycorrhizaFinder: a user-friendly machine-learning tool to quantify endomycorrhizal colonisation of nursery and field roots

**DOI:** 10.1007/s00572-026-01323-x

**Published:** 2026-10-23

**Authors:** Jill Kowal, Shaun Buckley, Robin Upham, Adam Kiani, Morgan Rickards, Ella Serpell, Martin I. Bidartondo, Edouard Evangelisti, Sebastian Schornack, James Sibbit, Kevin P. Treder, Simon Weidinger, Laura M. Suz

**Affiliations:** 1 Ecosystem Stewardship, Royal Botanic Gardens, Kew, TW9 3DS England; 2 Natural History Museum, London, SW7 5BD England; 3 Imperial College London, Ascot, SL57PY England; 4 Institut Sophia Agrobiotech, UMR INRAE 1355, Université Côte d’Azur, Sophia Antipolis, Provence-Alpes-Côte d’Azur, 06103 France; 5 Sainsbury Laboratory, University of Cambridge, Cambridge, CB2 1LR England; 6 D-fine GmbH, 60313 Frankfurt, Germany; 7 Institut Botànic de Barcelona (IBB), CSIC-CMCNB, Barcelona, 08038 Spain

**Keywords:** Arbuscular mycorrhiza (AM), Dark septate endophytes (DSE), Ericoid mycorrhiza (ErM), Root colonisation, Artificial intelligence (AI), Ecosystem assessment

## Abstract

Root colonisation by endomycorrhizal fungi has long been a widely used metric in mycorrhizal studies. However, due to the significant time required to assess colonisation using traditional microscopy techniques, studies of colonisation at large scales are impractical. Machine-learning-based approaches may increase output and facilitate ecosystem assessments. We trained a machine-learning-based tool (MycorrhizaFinder) on 42 field roots (132 unique 1 cm sections, with > 100,000 tiles) and validated on a further 37 sections from grasslands and heathlands hosting common Northern European plants with arbuscular (AM) and ericoid mycorrhizal (ErM) fungal structures, and dark septate endophytes (DSE), also common in field-sourced roots. We incorporated a user-customized confidence threshold to encourage engagement with morphological ambiguities, in e.g. ErM and DSE. A Macro F_1_ statistic was used to assess the tool’s development performance. We provide a sample workflow from root processing and microscope slide scanning to semi-automated model training and performance evaluation. Our model performance combining all classes, the Macro F_1_, is conservatively 66% for arbuscular and 57% for ericoid mycorrhizal colonisation assessment. MycorrhizaFinder is user-friendly, requires no programming skills and offers flexibility for advanced agronomists or ecologists to train the tool using their own labelled mycorrhizal root datasets, including images acquired from different instruments or staining protocols. This adaptability allows users to customize the model for specific ecosystems or experimental designs. Leveraged with molecular identification and/or functional traits of fungi (e.g. arbuscules), MycorrhizaFinder supports scalable and repeatable nursery experiments and monitoring across ecosystems to assess mycorrhizal status and track land-use changes over time.

**Supplementary Information:**

The online version contains supplementary material available at https://doi.org/10.1007/s00572-026-01323-x.

## Introduction

Most land plants form endomycorrhizas, defined by nutrient exchange between a plant root (‘host’) and beneficial fungi (Brundrett and Tedersoo 2018). Roughly 85% of plants are colonised by intracellular hyphae of diverse Glomeromycotina and/or Mucoromycotina (Endogonales) fungi, forming arbuscular mycorrhizas (AM) or AM-like symbioses, with each subphylum having distinct morphological and nutritional functional traits (Hoysted et al. 2018, 2019). Ericaceae plants found in Northern European heathlands and abundant as understory in woodlands are instead colonised by ericoid mycorrhizal (ErM) fungi. The Ericaceae constitute ~ 1.5% of terrestrial hosts for members of the fungal phyla Ascomycota and Basidiomycota. Many ErM types have been described since the classical intercellular ascomycete coils were described by Read (1996); Kiheri et al. (2020) describe four ErM fungal taxa, distinguishing them from dark septate endophytes (DSE), a non-mycorrhizal group of fungi which have more heavily melanised septate hyphae. The ecological functions of DSE remain enigmatic (de Oliveira and Pereira 2025), but they are globally widespread and may be central to understanding some ecological systems (Netherway et al. 2024). A continuum of structures has also been observed between ErM and DSE in e.g. *Rhododendron* spp., drawing attention to both the morphological ambiguities that may arise when categorizing mycorrhizal type based on morphology alone, and the unresolved biological functions of both groups (Vohník and Albrechtová 2011).

Although the physiological significance of mycorrhizal colonisation in nature has been questioned and presents some uncertainties, e.g. the percentage of root length colonisation can predict intra-radical colonisation for some taxa but not all (Hart and Reader 2002), it can contribute to explain differences in AM abundance across diverse sites (Jansa et al. 2019). Arbuscular mycorrhizal richness can correlate with root colonisation assessments comparing farm management types (Verbruggen et al. 2012), and more directly, the extent of mycorrhizal colonisation can be an informative, context-dependent indicator of functional outcomes for host plants, responsive to edaphic changes and host secondary metabolites (Soudzilovskaia et al. 2015; Frew 2025). Using measures of mycorrhizal colonisation, a recent field study showed host-mediated interactions between AM and saprotrophic fungi can drive soil organic carbon dynamics (Tang et al. 2026) and AM species richness. Positive relationships between AM and ErM fungal colonisation levels with aboveground growth and resilience in field and laboratory experiments have been reported (Diaz et al. 2006; Kowal et al. 2016; Lethielleux-Juge 2025; Frew et al. 2025). A habitat scale study showed that AM root colonisation and soil neutral lipid fatty acids were strongly correlated, raising the possibility of using colonisation as a proxy for soil AM biomass (Barcelo et al. 2020). Typically, colonisation is evaluated using microscopy, either by counting a number of grid intersections with colonised root fragments (5–10 mm) using a microscope eyepiece reticle, or by estimating the proportion of root cells that are colonised (McGonigle et al. 1990; Kowal et al. 2020). While these approaches offer reasonable objectivity and reproducibility, they are labour-intensive and therefore impractical for ecosystem-scale assessments, which often require the evaluation of hundreds to thousands of root samples. Despite increasing knowledge of AM lifestyle tendencies, i.e. rhizophilic or edaphophilic (Odriozola et al. 2026), and their different nutrient roles (Jenab et al. 2026), there remains a need for a standardised method for colonisation assessment of field roots to allow further investigation of the physiological and ecological significance of colonisation in nature.

As part of the UK Department for Environment, Food and Rural Affairs’ (Defra) England-wide Natural Capital and Ecosystem Assessment (NCEA) programme, we have been evaluating field-sourced roots for a nationwide baseline assessment of mycorrhizas across terrestrial habitats, including arable and horticultural land, grasslands, moorlands, woodlands, heathlands and bogs. To expedite the analysis of thousands of roots, we initially tested the suitability of AMFinder (Evangelisti et al. 2021), a tile-based computer vision tool using a shallow convolution neural network (CNN) architecture developed to quantify AM in four species of laboratory grown model-plant seedlings (*Nicotiana benthamiana*,* Medicago truncatula*,* Lotus japonicus* and *Oryza sativa*). The mycorrhizal fungal symbionts introduced to colonise these roots were from monoxenic cultures of four AM genera - *Rhizophagus*,* Claroideoglomus*,* Rhizoglomus* and *Funneliformis*. The authors used three categories or classes to distinguish tiles (colonised, uncolonised and non-root) and predict mycorrhizal colonisation. AMFinder produced highly accurate predictions with high recall and precision (see Glossary) using these seedling roots. However, when tested with our high-resolution images of field-sourced root sections cleared and stained with similar ink-vinegar protocols, it often could not recognise roots, and only performed well, i.e. with an error rate of 20% or less, in rare cases where hyphae were well-defined and roots were dominated by a single Glomeromycotina or Mucoromycotina (Endogonales) morphotype. Field roots usually present a complex mosaic of AM or ErM types depending on host and fungal life stage, often with irregular hyphal diameters, and co-occurring with DSE and other non-mycorrhizal fungi, microsclerotia and bacteria. More advanced image analysis techniques developed to analyse AM colonisation, including deep learning or computer vision models, have so far only been trained on a limited set of plants such as crops (e.g. sorghum or soybeans) grown and inoculated in controlled settings (Kokkoris et al. 2019; Muta et al. 2022; Sciascia et al. 2023; Zhang et al. 2024).

Overall, despite the increasing availability of computer vision tools for rapid analysis of mycorrhizal colonisation, existing solutions rely on root images from controlled nursery or laboratory experiments with limited variation in host plant, fungal symbiont or level of disturbance of roots. This creates a significant gap in efforts to accelerate colonisation assessment of field-collected roots, which are far more complex than binary plant-fungus samples, exhibiting diverse ecological backgrounds, variable staining quality and a mixture of fungal structures. To address this gap, we developed MycorrhizaFinder (MF, available at https://doi.org/10.5281/zenodo.17979413), a computer-vision tool to assess the colonisation of field roots across plant species and endomycorrhizal types, able to differentiate common artifacts and disturbance arising from field collection (e.g. root image obstruction by clay particle debris), and for use across terrestrial ecosystems. Our main aim was to investigate and test appropriate model architectures which would allow us to: (1) add additional classes in the classification of AM; (2) develop an alternative class model infrastructure focusing on ErM fungal colonisation that could be toggled within the same, unified user interface; (3) incorporate contextual information into the classification of each tile using the surrounding tiles; and (4) upgrade the user interface with all modelling, labelling and training streamlined in a single workflow with human supervision. Most significantly, MF aims to be accessible by researchers with no coding skills and for laboratory and field-sourced roots alike, paving the way for large-scale assessments. We do not intend to simplify the rich and diverse fungal morphological and ontogenetic still-life images shared in the accompanying dataset by distilling them into a handful of categories. Rather, we present a semi-automated machine-learning framework by which the researcher, e.g. biologist, ecologist or agronomist, can with high confidence settings, filter-out most uncolonised root cells and typical AM and ErM forms from scrutiny, allowing more time to analyse ambiguous fungal structures.

## Materials and methods

### Roots and images

**Fig. 1:**
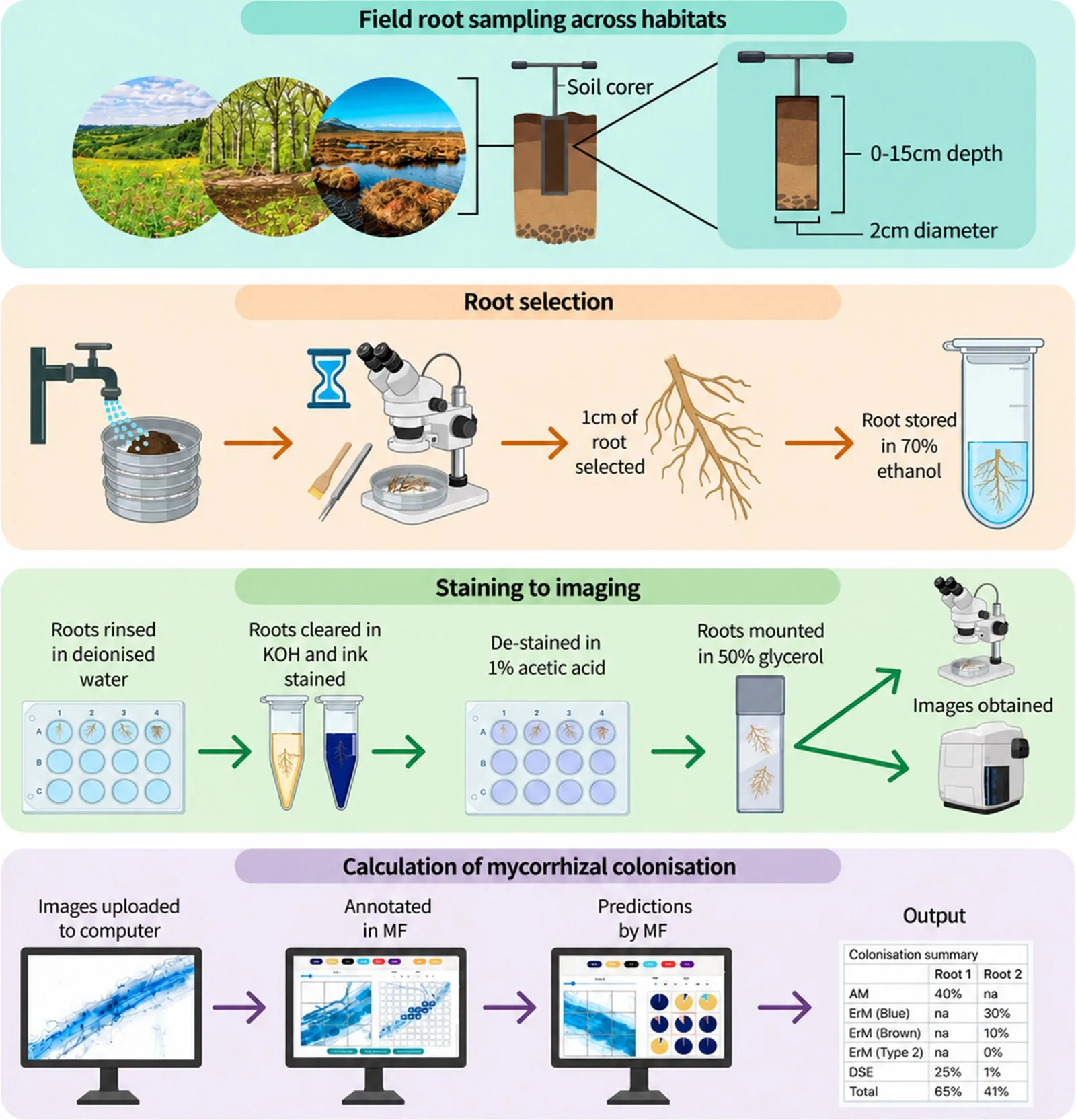
Workflow diagram showing field sampling, root selection, staining, imaging, and calculation of mycorrhizal colonisation. Bottom panel shows a simplified workflow of MycorrhizaFinder, from left to right: Image upload; Annotation of root section with nine tiles (training or editing); Predictions of tile class (pie-charts) with target tile outlined in red; Output, root level colonisation summary. Roots’ colonisation predictions can be processed in batches. Refer to https://doi.org/10.5281/zenodo.17979413 for further details

The roots used for training the machine-learning model were obtained from field collected soil cores (15 cm deep, 2 cm wide) from agricultural fields, grasslands, heathlands and bogs across England, and *Calluna vulgaris* and *Erica tetralix* roots from sites in Norway. Soil samples were placed in a metal sieve (0.5 mm aperture size) and rinsed in tap water. Non-woody fresh roots were selected to yield a diverse range of root architectures, from straight to fine with complex branching (Fig. 1). All roots from putative AM hosts were stained in blue-ink and mounted on slides by modifying Kowal et al. (2020) (see Methods S1). Trypan blue solution (0.05% v/v) was used for ErM-host roots as we hypothesized that the ink may be less effective at adhering to intraradical hyphae. Root sections for both putative-AM and ErM hosts were 1 cm long with variable surface according to root architectures. The slides were scanned to produce high resolution images using an Epredia 3DHISTECH Pannoramic MIDI II Rx brightfield digital slide scanner at 37x magnification (0.27 μm/pixel), but the tool can also adapt to JPG images acquired using a digital microscope (Evangelisti et al. 2021). To capture the three-dimensional, complex fungal network within the root cortex, an extended focus imaging approach was used to condense multiple-focal planes. Seven focus levels were used, where one step was around 0.2 μm. In total, root sections measuring approximately 1 cm in length from 132 unique roots (47 AM colonised, 85 ErM colonised) were selected for training after evaluating image quality and maximising quantity of tiles across classes (Table 1; complete training image library available at https://doi.org/10.5281/zenodo.17476686). Depending on the root, there were hundreds or thousands of tiles per root tissue area, i.e. non-background tiles, with each tile containing 252 × 252 pixels for AM and 126 × 126 for ErM tiles, set to align with typical root cell sizes.

**Table 1:** Summary of tile data classes used for training. Note: since a single root can contribute tiles from more than one class, the total number of roots used is less than the sum of the number of roots used for each class. AM: arbuscular mycorrhiza; DSE: dark septate endophyte; ErM: ericoid mycorrhiza

Model	Tile class/Abbreviation	Number of roots	Number of tiles	Percent of model tiles
Arbuscular mycorrhiza	AM Colonised/AM^+^	45	3,604	4.2
	DSE Colonised/DSE	34	808	0.9
	AM + DSE Colonised/H	24	276	0.3
	Uncolonised/N-	45	15,239	17.9
	Background/X	46	63,736	74.7
	Unreadable/U	37	1,705	2.0
Ericoid mycorrhiza	Blue Coils Colonised/Bl+	76	2,344	2.8
	Brown Coils Colonised/Br+	76	2,485	3.0
	Type Two Colonised/T+	9	319	0.4
	DSE Colonised/DSE	43	1,916	2.3
	Mixed ErM Colonised/HE+	56	314	0.4
	ErM + DSE Colonised/HD+	9	37	< 0.1
	Uncolonised/N-	85	6,492	7.9
	Main Root/M	85	12,838	15.6
	Background/X	88	50,564	61.3
	Unreadable/U	80	5,242	6.4

We also evaluated the model on a subset of images used to train AMFinder (but not MF) to test how the platform performed with images captured using a digital microscope more typical of plant science laboratories. We selected 19 root images compatible with the tile size parameters.

### Distinction and quantification of mycorrhizal structures

To more predictably estimate mycorrhizal colonisation in field roots, we created more classes than the original three-class AMFinder (Evangelisti et al. 2021). This allowed our machine learning model to recognise a wider range of real-world root and fungal biological features than in the original training images used to build AMFinder. Real-world field roots present variable hyphal diameters and textures within diverse root architectures, co-occurrence of DSE, and broken cell wall noise from soil extraction. We modified the two original CNNs (CNN1 and CNN2) to expand the CNN1’s output layer from three classes in AMFinder to six in our model. Moreover, the CNN2’s functionality was incorporated into CNN1 for the ErM mode path, allowing it to differentiate between hyphal structures, described in Table 2. Images were augmented and datasets’ classes balanced using several standard machine learning techniques (see Methods S2).

**Table 2:**
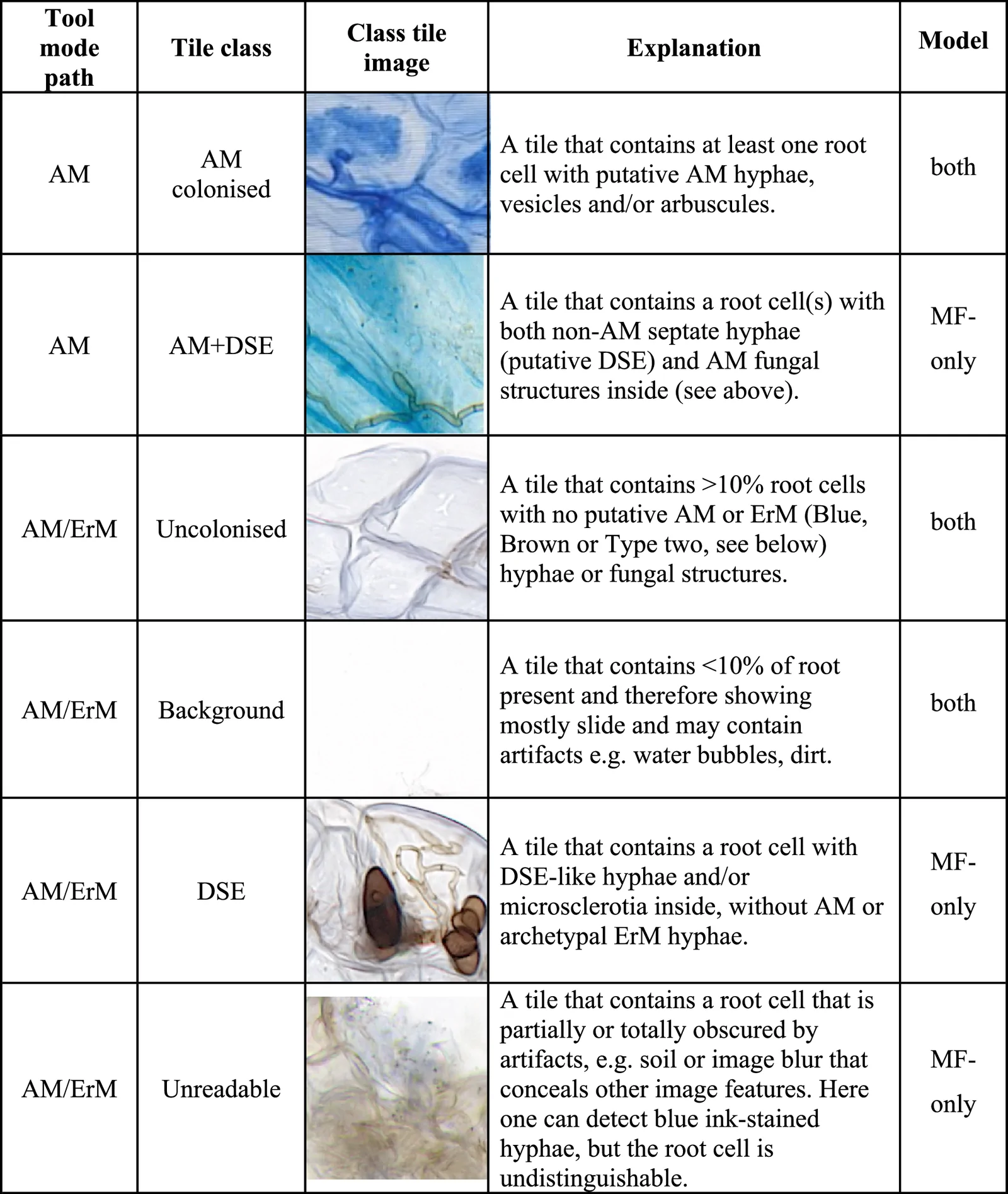

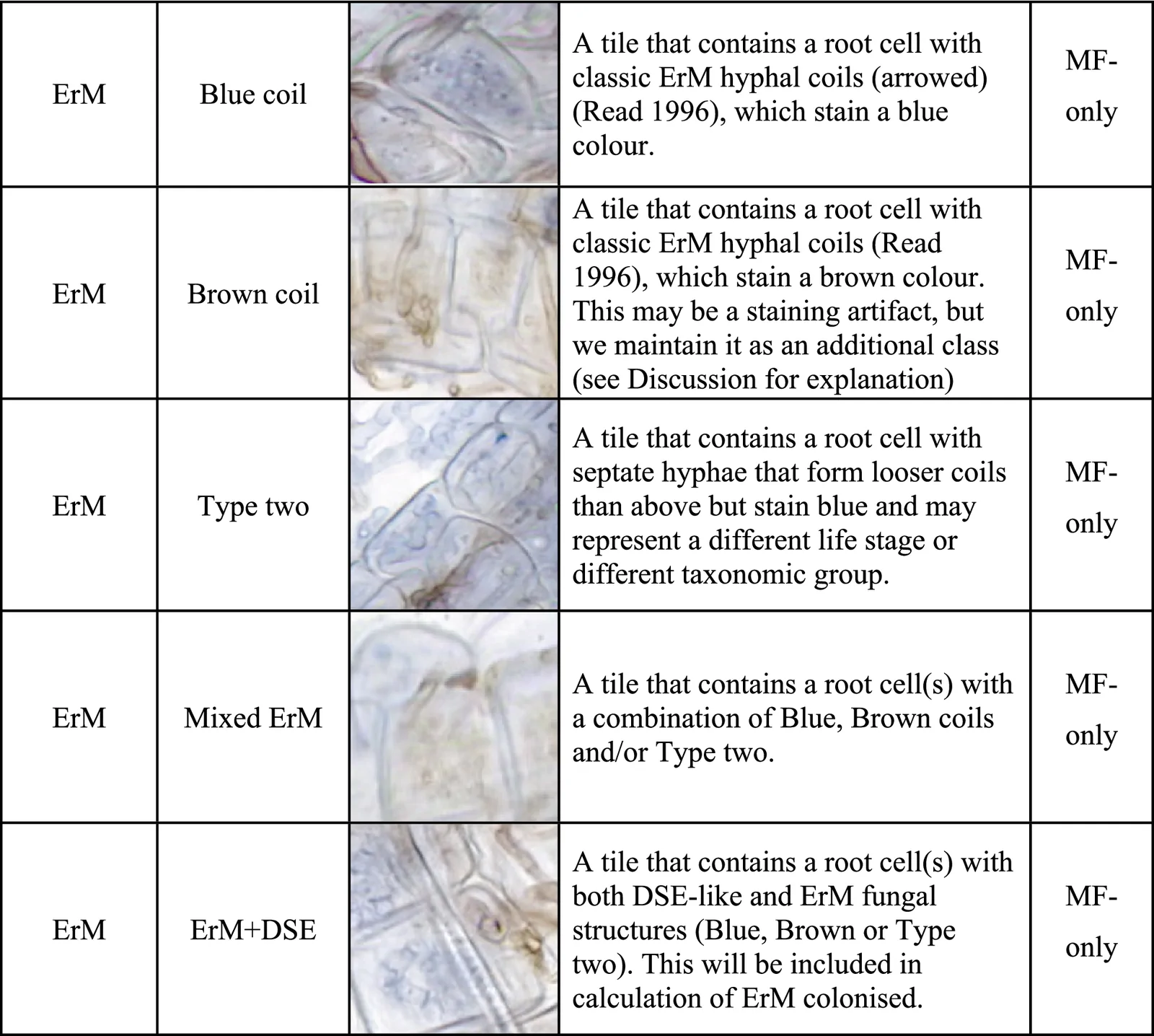
Tile classes utilised in the model output layer of AMFinder (Evangelisti et al. 2021) and MycorrhizaFinder(MF). Either ‘both’, AMFinder and the expanded MF models, or ‘MF-only’ for new features of arbuscular mycorrhiza (AM) and ericoid mycorrhiza (ErM) detection in roots. Class tile images show examples of defined class feature(s)

To accommodate a greater number of classes, we evaluated four model architectures - ResNet, ResNeXt, the AMFinder ‘CNN1’ model and EfficientNetB5 (Tan and Le 2019) - and the latter was ultimately selected for further analysis (see Methods S2). Model training and testing were also conducted using the original CNN1 architecture to establish a benchmark for comparative performance evaluation.

The dataset breakdown of tiles by class is presented in Table 1. There may be more than one root cell within a tile or a partial cell, as long as the cell walls are distinguishable and cover 10% of the tile (see MycorrhizaFinder Full User Documentation, https://doi.org/10.5281/zenodo.17979413).

A new class structure for Ericaceae plant family roots was added. We differentiated three ErM colonisation types based on morphology (*Blue coils*, *Brown coils* and *Type two*) (Table 2), for potential future analysis exploring biological and functional differences, recognising that these do not capture all the possibilities including a ErM-DSE continuum and hyphae related to ‘sheathed’ ErM host structures (Vohnik et al. 2012), which appear similar to Type two, but cannot be confirmed with cytological observations alone, i.e., without the whole-root context. We also distinguished ‘Main Root’ as the colonisation calculation is based only on young non-lignified roots.

### Quantification of mycorrhizal colonisation

We programmed the model to predict root colonisation in two steps. First, the model processes one tile at a time and computes the probability that the tile belongs to each class. Then, the contextual mode (default) incorporates four neighbouring tiles (with a diagonal overlap extending 75% from the central tile, Fig. S1) and uses a max-vote system to assign the class to each tile by combining majority predictions for all four neighbouring tiles with the target tile, thus generating a (new) class label for the central tile. As tiles and plant root cells are rarely aligned, this captured broader spatial context across neighbouring regions, mimicking the classification behaviour of a human when contextualizing observations, in this case to enable recognition of fungal structures within a complete root cell. Further details of the context feature are in Methods S2.

We included a feature allowing the user to switch on a threshold of confidence (the probability of the most probable class) above which, this context feature is disabled on a per-tile basis to save computing time. It defaults to a value of 1 as there is a systematic increase in overall predictive performance with the contextual feature enabled.

Once predictions are generated, they are required to be converted to annotations (definite class labels) to calculate the colonisation metrics. At this stage it is again possible to supply a threshold value of confidence, above which predictions are automatically converted to annotations of the most probable class and below which predictions are ignored. To solicit user recognition of the ambiguities inherent in these groupings, we suggest the user start at 80% (see user guide).

The calculation of colonisation is explained in Table 3. Each colonisation metric was defined as the ratio of tiles designated as belonging to a class(es), relative to the total number of tiles belonging to all root classes, excluding tiles which were background or unreadable.

**Table 3:** Definitions of mycorrhizal fungal colonisation metrics calculated by MycorrhizaFinder. The name of each class (*AMColonised*, (*AM + DSE*), etc.) refers to the number of tiles in the image belonging to the relevant class. For arbuscular mycorrhiza (AM), *TotalRoot* is defined as *Uncolonised* + *AMColonised* + *DSE* + (*AM + DSE)*; for ericoid mycorrhiza (ErM), it is defined as *Uncolonised* + *BlueCoils* + *BrownCoils* + *TypeTwo* + *DSE* + *MixedErM +* (*ErM + DSE).*

Colonisation type	Colonisation metric	Metric equation
Arbuscular mycorrhiza	Arbuscular mycorrhiza (AM)	$\:\frac{AMColonised\:+\:(AM+DSE)}{TotalRoot}$
	Dark septate endophyte (DSE)	$\:\frac{DSE\:\left(only\right)}{TotalRoot}$
	Total	$\:\frac{AMColonised\:+\:DSE\:+\:\left(AM+DSE\right)}{TotalRoot}$
Ericoid mycorrhiza	BlueCoils (Bl)	$\:\frac{BlueCoils}{TotalRoot}$
	BrownCoils (Br)	$\:\frac{BrownCoils}{TotalRoot}$
	Type two	$\:\frac{TypeTwo}{TotalRoot}$
	Dark septate endophyte	$\:\frac{DSE\:+\:(ErM.DSE)}{TotalRoot}$
	Total ErM colonisation	$\:\frac{Bl\:+\:Br\:+\:TypeTwo\:+\:MixedErM+(ErM+DSE)}{TotalRoot}$

### Performance of the model efficacy with colonisation assessments

We quantified performance using an *F*_*1*_ Score (see Eq. 1) at the root level, which calculates the harmonic mean of *Precision* and *Recall* (see Glossary) yielding a value between 0 and 1, where 1 indicates the best performance (Chinchor 1992; Grandini et al. 2020; Reinke et al. 2021; Fergus et al. 2022). There are two equivalent definitions, either using *Precision* and *Recall* or the number of true positives (*TP*), false positives (*FP*) and false negatives (*FN*):

1 $$\:F_1=\frac{2\;.\:TP}{2\;.\:TP+FN+FP}=2\;.\:\frac{\left(Precision\;.\:Recall\right)}{\left(Precision+Recall\right)}$$

The *F*_*1*_ Score penalises extreme values of either *Precision* or *Recall* and rewards models having similar high values for both, whilst simultaneously being robust against imbalanced datasets (Fergus et al. 2022, Khan et al. 2021, Crespo-Michel et al. 2023; Rahman et al. 2023). This was a key advantage over the *Accuracy* measure (see Glossary) originally used to quantify the predictive performance of AMFinder in Evangelisti et al. (2021). We applied an *F*_*1*_ Score to each individual class and across the entire model as *Macro F*_1_, thus incorporating corresponding *Precision* and *Recall* scores across all classes (Fig. S2) (see Eq. 2, Opitz 2024).

2 $$\:Macro\:F1=\frac{1}{n}\:\sum\:_{i=1}^{n}{F1}_{i}$$

### Development of a user-friendly interface

To improve user experience and accessibility for non-computer experts we: (1) streamlined the way the user interacts with the application, by allowing the user to access all the tool functionalities including training via a single user interface (Fig. S3; see Methods S2 for further details on model training); (2) upgraded the user interface to encapsulate the end-to-end workflow in the tool, from uploading TIFF-format slide images and splitting them into smaller JPGs for utilisation by the tool, to calculating predictions and outputting colonisation percentages (at the root level) with uncertainty measures, thus simplifying the workflow to allow all activities to be carried out via a centralised point of access and decision making; (3) added a confidence threshold setting to the existing workflow to ensure users can review uncertain predictions and overwrite them manually if needed. This modification ensured that there was a human in the loop for assessing the output of the machine learning model and enabled users to balance automation with certainty; (4) added high-level image overviews for reference while annotating and included the ability to save annotations and maintain unknowns temporarily (using ‘?’) without needing to close the tool. This function was intended for less experienced analysts allowing them to optionally embed comments and proceed with other annotations while awaiting expert supervisors input on uncertain tiles; (5) migrated the user interface from a standalone application written in the OCaml programming language to a more versatile web-browser-based application written in *React*; (6) replaced the data storage system of zipped CSVs used in AMFinder with a PostgreSQL database for storage of predictions and annotations, aligning with industry standards and improving data governance; and (7) added a persistent configuration to the tool to allow users to save settings between sessions (for example whether to use contextual mode when generating predictions).

## Results

### Quantification of AM fungal colonisation in field-collected roots using MF

**Fig. 2:**
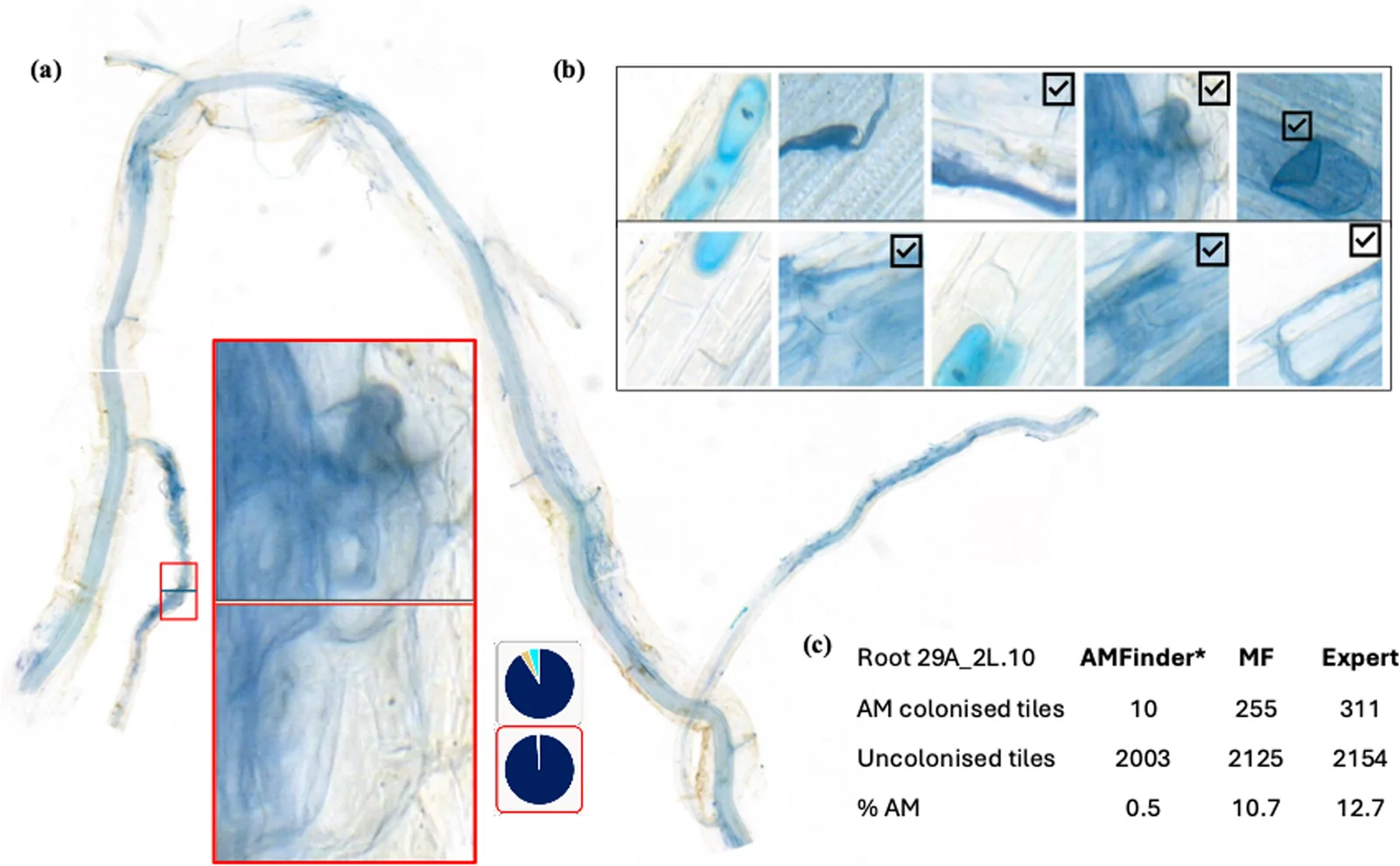
Comparison of colonisation predictions using the original AMFinder and MycorrhizaFinder (MF) on the same arbuscular mycorrhizal (AM) field-sourced root after clearing, staining and high-resolution scanning. **a**) Image root section (1 cm in length), showing example of two colonised tiles (red-lined boxes) that were not counted as colonised by AMFinder (i.e. false negatives) but were correctly predicted by MF. AM colonised is represented by dark blue in corresponding pie-charts. **b**) The ten tiles predicted as AM colonised by AMFinder, black tick boxes on 6 of 10 tiles indicate predictions are expert validated leaving 4 of 10 as false positives. **c**) Summary of tile counts and colonisation predictions generated by AMFinder versus MF for this root are significantly different. ‘% AM’ is percent of root tiles presenting any tile class with AM (see Table 2). Human expert analysis of tiles is included for contrast

Automated predictions of colonisation improved significantly compared with the original AMFinder tool as seen in the calculation of colonisation using the automated function on independent test roots (Fig. 2). By adding manual annotation to these (in tiles beneath the set confidence threshold), accuracy on par with microscopic examination was achievable.

AMFinder repeatedly misclassified field root tiles. In the example shown in Fig. 2b, four of the ten tiles predicted as AM were incorrect due to their biological ambiguities. Overall, MF achieved significantly better detection of AM class tiles resulting in prediction of 10.7% AM colonisation compared with 0.5% for AMFinder, before human augmentation, and despite biological and technical artefacts e.g. patchy staining, soil debris. Expert estimates of colonisation were 12.7%. See Figure S4 for additional examples of MF compared with a human expert; overall differences in the AM-related classes were minimal.

### Incorporating contextual information significantly improves prediction quality

**Fig. 3:**
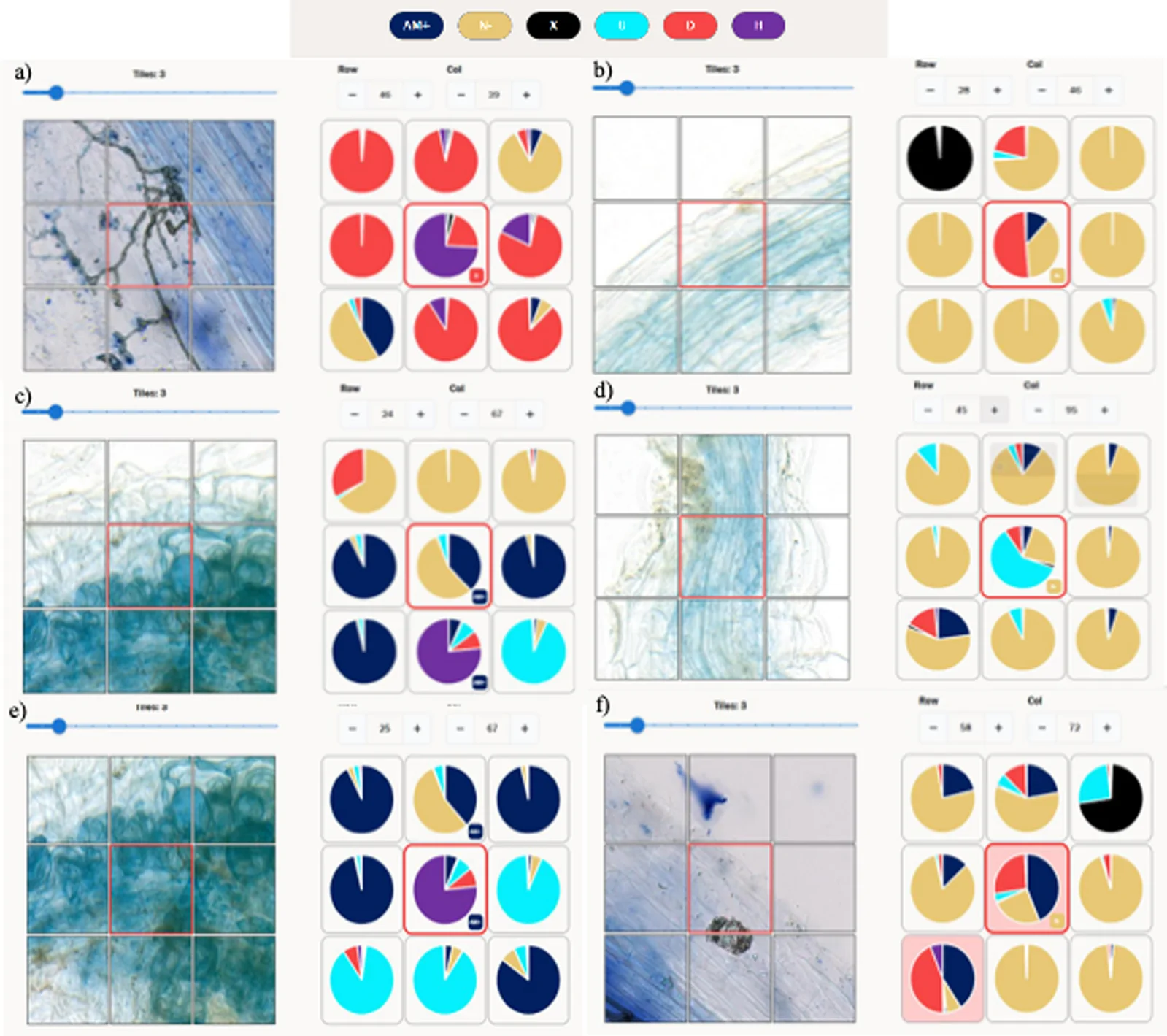
Examples of tile classes that switch when applying the contextual function in MycorrhizaFinder for the arbuscular mycorrhiza (AM) pathway. The class to which it changes is shown in the lower right corner of the central, red-bordered target tile. Tile class designation before and after context implementation for the six examples are detailed here: (**a**) AM + DSE (H) to DSE (D); (**b**) DSE to Uncolonised (N-); (**c**) Uncolonised (N-) to AM Colonised (AM^+^); (**d**) Unreadable (U) to Uncolonised (N-); (**e**) AM + DSE (H) to AM Colonised (AM^+^); (**f**) AM Colonised (AM^+^) to Uncolonised (N-). See Table 2 for tile class descriptions, and Fig. S1 for an illustration showing the extent of overlap by neighbouring tiles when activating the context function

**Fig. 4:**
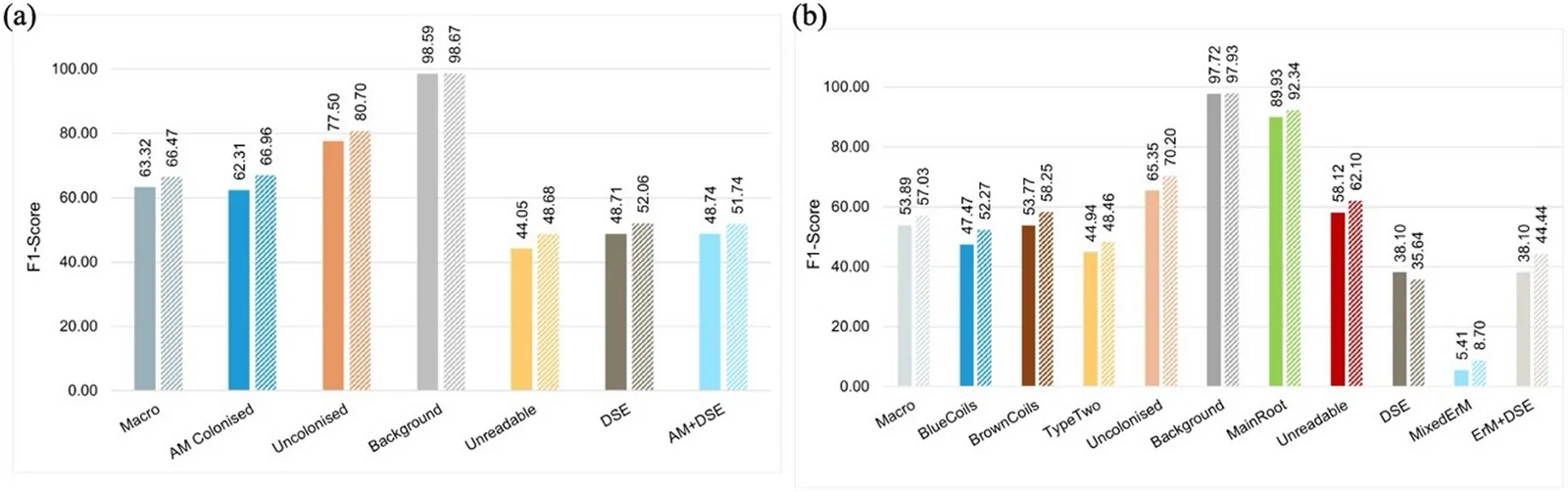
Performance (*F*_*1*_ Score) of MycorrizaFinder with and without the context feature (lighter and darker paired colour bars, respectively). (**a**) The arbuscular mycorrhiza (AM) path on our test dataset of AM host roots. (**b**) The ericoid mycorrhiza (ErM) path on our test dataset of ErM host roots. *Macro* reflects the *F*_*1*_ Score (Eq. 2) for the entire model averaged over all classes; the other bars refer to the individual *F*_*1*_ Scores of the corresponding classes. Scores are expressed as a percentage, where the maximum score is 100. Scores have been obtained on the same model weights with all confidence thresholds set to 1, without any human supervision. See Table 2 for tile class descriptions

Tile-level predictions on an AM-colonised root region with and without the contextual function can yield significantly different determinations of class, affecting root-level colonisation percentages (Fig. 3). We observed a consistent improvement of the mycorrhizal class performance function when activating the contextual function, independent of the model architecture (Fig. 4). The *F*_*1*_ Score for all classes improved with context using both the AM and ErM pathways (except for *DSE*), while the overall *Macro F*_*1*_ improved by 5.0% for AM and 5.8% for ErM. Combining the model architecture and context function changes, the *Macro F*_*1*_ improved significantly by 44.9% and 40.4% for AM and ErM, respectively, when compared against AMFinder (e.g. compare *Macro F*_*1*_ in Fig. 4 with Fig. S1).

In the AM case (Fig. 4a), the highest class *F*_*1*_ Score was consistently *Background*, with values over 98 (both with and without context), followed by the *Uncolonised* achieving 77.5 (non-contextual) and 80.7 (contextual). These two classes were also the most represented in the training dataset (Tables S7 and S8), with corresponding relative shares of 74.7% and 17.9%. However, balancing measures applied in the pre-processing steps during the training stage reduced their actual number to the count of the *AM Colonised* “anchor class”, which the other model classes were measured against (Methods S2). With the ErM dataset (Fig. 4b), the highest *F*_*1*_ Scores were observed for the classes *Background*, *Main Root* and *Uncolonised*, attaining 97.7, 89.9 and 65.4, respectively.

For both datasets and models, the application of the contextual function improved the *F*_*1*_ Scores in most classes, with few exceptions. This also held true for the shallower CNN1 model architecture (Figs. S3a, b).

### The F1 Score provides a robust and interpretable estimate of colonisation accuracy

We found that the additional tile classes enabled better recognition of mycorrhiza structures, (see *F*_*1*_ Scores, Fig. 4). Differentiating tiles with DSE and other artifacts typical of field roots, allowed the machine learning to focus on the mycorrhizal classes.

**Fig. 5:**
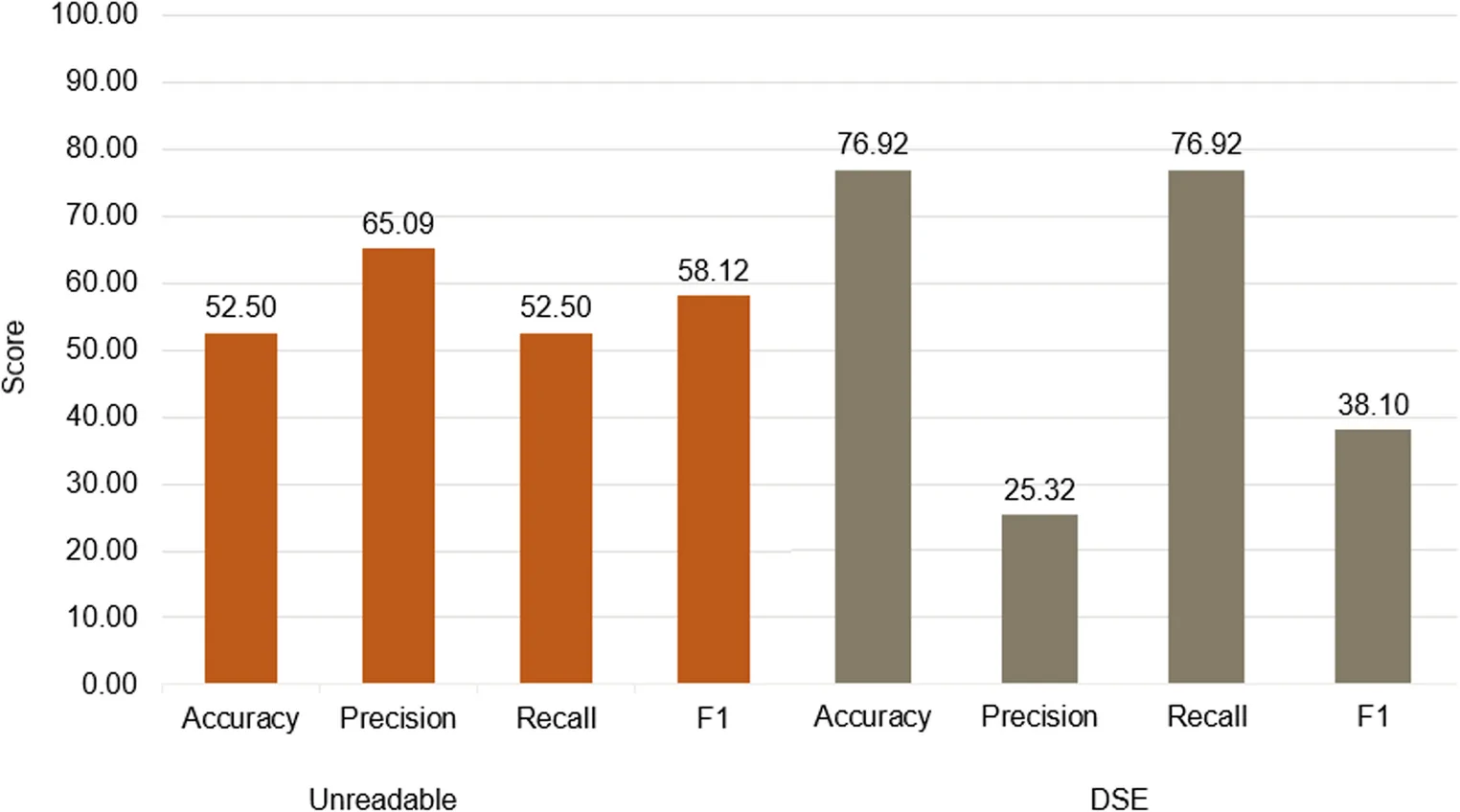
Quality metrics for the underrepresented classes ‘Unreadable’ and ‘DSE’, as measured on an independent test set for the ErM data and using the deployed EfficientNetB5 model (without the context feature). Calculations of *Accuracy* and *Recall* for the Unreadable and DSE classes are the same in this case. See Table 2 for tile class descriptions

Classes with higher sample representation yielded a higher contribution to the model *Accuracy* than classes with lower representation, as seen in the performance of the ErM model (no context) for an independent test set of the classes ‘Unreadable’ and ‘DSE’ (Fig. 5). The *Macro F*_*1*_ not only gave all classes the same weight towards a single performance measure (independent of their prevalence in the dataset) but also provides clarity on whether all samples for a given class were captured (*Recall*) and actually correct (*Precision*). While the *Accuracy*_*DSE*_ was measured at 76.92 on the independent ErM test dataset, suggesting a high performance if this was the only used metric, the corresponding model *Precision*_*DSE*_ was measured at 25.32, leading to a class *F*_*1*_ Score_*DSE*_ of 38.10. Inversely the *Accuracy*_*Unreadable*_ of 52.50 suggested lower performance, if it was the sole component of the metric. The corresponding *Precision*_*Unreadable*_ of 65.09 raises the total *F*_*1*_ Score_*DSE*_ to 58.12.

### MF facilitated quantification of ErM fungal colonisation in field-collected roots

**Fig. 6:**
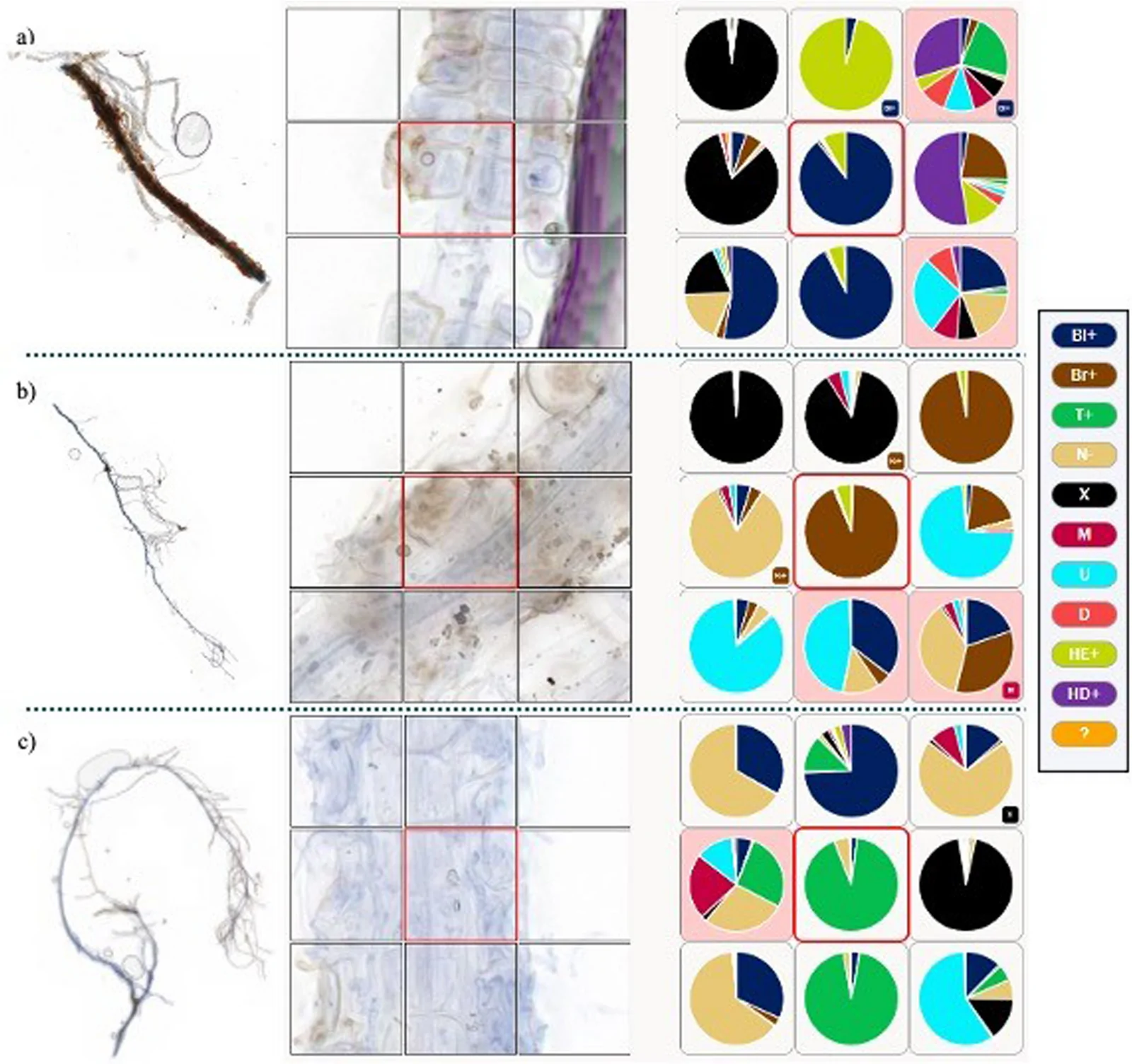
Representative images of three ericoid mycorrhizal (ErM) field roots and class predictions using the MycorrhizaFinder ErM pathway. Central images show a nine-tile panel magnifying a section of root. The pie-charts show corresponding class predictions, with the contextual function switched on. The legend details one of ten class possibilities (see Table 1 for reference of tile class abbreviations). The central, red-bordered image tiles were predicted as (**a**) *Blue coils* (Bl+); (**b**) *Brown coils* (Br+); and (**c**) *Type two* patterns (T+). A small box label in the lower right corner of the classification tile indicates that the context function yielded a different outcome and amended the tile prediction. A classification tile with a red background indicates that the predictions were below a set conversion threshold (default 0.5) pointing the user to manually annotate

The ErM pathway was able to automatically distinguish features across the ErM classes *Blue coils*, *Brown coils*, *Type two* and *MixedErM/ErM + DSE* most of the time, and performed better with context. Representative examples of the tool distinguishing amongst biological features and artifacts (without human corrections) are shown in Fig. 6. The central tile (Fig. 6a) predicted Bl+ while the tile above initially predicted HE+, but with context, corrects to Bl+. In Fig. 6b the model predicted the central tile Br+, with high confidence, while the tile to its right is predicted U. The lower row yielded two of three tiles with pink backgrounds, showing that the threshold of certainty for all potential classes was below the conversion threshold (here set to 0.5). This shows how the programme signals to the user that a manual annotation is required if the tile is to be included in the calculation of colonisation metrics. In Fig. 6c the panel shows the ability of the model to distinguish the thicker T+ hyphae from the more common Bl+ coils.

A dedicated ErM model path significantly improved the biological ambiguity in distinguishing ErM septate hyphae structures from AM aseptate hyphae in roots from complex field environments. With the AM path, ErM structures were not typically misclassified as AM fungi, but commonly misidentified as DSE, thereby underestimating mycorrhizal colonisation (see Table S4). Our model development showed that with more training data, the individual *F*_*1*_ Scores continue to improve.

### Throughput was faster and simpler

Automated predictions of root colonisation using MF, as with AMFinder, significantly reduced analytical time compared to traditional techniques involving a human using a microscope. By allowing to set the confidence threshold, the user manages whether more or fewer tiles require expert input (Fig. S5). As the user changes the confidence settings e.g. 50%, 60%, 70%, 80%, 90%, the mean absolute error in colonisation estimates is consistently around 5% (Table S5). Also, with MF, augmentation of classes by user supervision was greatly simplified by allowing modifications of tile classifications within the same interface, without exiting and reloading, which was necessary previously. Single root images are now loaded in one step without splitting into several JPGs (see Fig. S6) as the tool can now manage significantly higher resolution JPGs than previously. Moreover, if necessary, conversion from TIFF to JPGs was also supported by the tool interface. Finally, the user no longer required programming skills to start-up the tool and could run it on a laptop on Windows, MacOS or Linux, albeit this is slower than using a dedicated desktop computer.

### Performance using other image capture devices

The MF model performed well on a subset of images (from the Evangelisti et al. 2021 dataset) captured using a high-resolution light microscope. The 3-class *Macro F*_*1*_ was 58% (without enhancements) compared with our trained roots of 65% (Fig. S7). Previously, the model with only three classes was wrongly predicting tiles containing AM vesicles and hyphae, or uncolonised cells as DSEs (Figures S8, S9).

### CNN1 model vs. EfficientNetB5

The EfficientNetB5 architecture (Tan and Le 2019) achieved high classification performance on a fully independent test dataset while being operational and retrainable on local GPUs (Table 4) and was ultimately chosen for the final tool deployment. Larger models, such as EfficientNetV2M (Tan and Le 2021) did not achieve significantly higher performance despite being built on a significantly higher number of parameters (EfficientNetB5 at ~ 30 M vs. EfficientNetV2L at ~ 54 M, Table S6).

**Table 4:** *Macro F*_*1*_ Scores for individual EfficientNetB5 (Tan and Le 2019) model architectures alongside the best CNN1 models as a benchmark comparison for the Arbuscular (AM) and Ericoid (ErM) Mycorrhiza datasets. The contextual function was not implemented for the AM CNN1 model

Dataset	Model Architecture	Macro F_1_
AM	CNN1 – without contextual function	45.88
	CNN1 – with contextual function	n/a
	EfficientNetB5 – without contextual function	63.32
	EfficientNetB5 – with contextual function	66.47
ErM	CNN1 – without contextual function	40.61
	CNN1 – with contextual function	42.28
	EfficientNetB5 – without contextual function	53.89
	EfficientNetB5 – with contextual function	57.03

There was a significant improvement of the *Macro F*_*1*_ Score when comparing the simpler CNN1 model to the EfficientNetB5 model for both the AM and ErM pathways (Table 4). The EfficientNetB5 model achieved higher classification performance when trained on the same dataset. Since both model architectures were optimised on the same training dataset, we concluded that the deeper model architecture of EfficientNetB5 captures complex image features in the dataset more effectively, leading to higher classification performance. This was also observed when comparing the individual class *F*_*1*_ Scores achieved in the CNN1-model with the ones obtained on the EfficientNetB5 model (compare Figures S10 with Fig. 4).

## Discussion

Standardised, medium to high throughput methods for mycorrhizal root colonisation assessments in field roots are needed by researchers, policy-makers and land managers to provide repeatable processes to monitor environmental change across scales. Such a method will facilitate direct comparison across different studies and ecosystems and facilitate understanding of the physiological and ecological significance of mycorrhizal colonisation in nature.

Our new MF tool addresses a research gap by presenting a computer vision tool specifically trained and optimised for field-collected roots successfully differentiating common artifacts and disturbances arising from field collection with different endomycorrhizal types (through the AM and ErM pathways). It has also significantly improved access for researchers to assess root colonisation for controlled nursery and laboratory experiments at scale.

By applying multiple technical innovations to AMFinder, our upgrades provide an enhanced user experience with an intuitive design. The portable *React* (Meta Open Source 2025) implementation results in a tool accessible to non-experts and adaptable across all major computer operating systems. The user interface allows human oversight on the predictions calculated by the model. Thus, expert tasks are reduced to assessing the machine predictions of the relevant tiles of each microscope image, with confidence thresholds determined by the user, thereby inviting trade-offs between certainty and time inputs root by root. The tool can be run on a single desktop computer without requiring advanced hardware, though prediction time is reduced when a dedicated GPU is available. Replacing the preexisting CNN1 model with a pretrained EfficientNet (Tan and Le 2019) architecture, allowed us to introduce new classes for AM assessments - *DSE*, mixed *AM +* DSE and *Unreadable* - to improve the performance of assessing colonisation by removing ambiguity in visual recognition. The higher classification performances of *Background* and *Uncolonised* was mainly due to less intricate image features compared to the other classes. Experiments and performance measures obtained on corresponding CNN1 models outlined a comparable classification behaviour (compare with Figs. S7, S8). The model’s lower *F*_*1*_ Scores in the cases involving classification of fungal structures (*AM Colonised*, *DSE Colonised* and mixed *AM + DSE Colonised*) signify higher complexity in image features compared to the *Background* and *Uncolonised* classes. Taking the relatively constrained shares of data into account (Table 1) i.e. 0.9% for the DSE *Colonised* class and 0.3% for the *AM + DSE Colonised*, the obtained *F*_*1*_ Score result was analytically meaningful and indicates the model architecture’s capacity to generalise. A similar reasoning could be applied when revisiting the results of the *Unreadable* class, adding that the image features encountered in this class were based on blurry, out-of-focus or indefinable diversity instead.

Our new model for ErM colonisation arose because the AM pathway, trained on aseptate fungi, is not suitable for recognising septate ericoid hyphae classes as evidenced by the example shown in Table S4. By training a separate path for Ericaceae roots, we are able to more efficiently evaluate Northern European Ericaceae-dominant habitats at scale. In most cases, the sample collector will know if the host is Ericaceae as these woody shrubs are apparent year-round. The model’s *Main Root F*_*1*_ performance is stronger than the ErM classes, providing confidence that the model is already able to distinguish the older more lignified root tiles leading to a more reliable estimate of colonisation. These values could be attributed to a similar rationale as in the case of the AM dataset i.e. image features encountered in the *Background* and the *Main Root* classes were more limited in their diversity and were easier to classify. A higher relative share in the training data could be excluded as these classes originally exhibited a higher level of representation compared to the *Blue Coils* “anchor class” and were thus balanced via clipping (Table S3, Methods S2). This was further supported by results obtained on the CNN1-ErM model (see Fig. S2c), where a comparable distribution in classification performance could be observed. The classes that characterise the colonisation of Ericaceae roots - *Blue Coils*, *Brown Coils*, *Type Two*, *DSE*, *Mixed ErM* and *ErM + DSE*, could be clustered into two performance groups. The first three achieved more robust *F*_*1*_ Scores around 50.0, which could be attributed to higher relative data shares (2.8%, 3.0%, 0.4%, respectively), and the latter three demonstrated greater variation in the obtained *F*_*1*_ Scores, which could also be attributed to relatively low representation in the training dataset (2.3%, 0.4% and < 0.1%, respectively). It appears however that the *ErM + DSE* class exhibited more recognisable image features compared to the *MixedErM* class, as a higher *F*_*1*_ Score was achieved despite significantly fewer images present in the data.

Given phenotypic complexities of ErM in field roots, by introducing new tile classes distinguishing different morphotypes within ErM (distinctly blue or brown colours and Type two), we aimed to be able to recognise potentially ecologically relevant signals occurring at the habitat scale. We were unable to conclusively attribute the blue and brown putative ErM to hyphal ontogeny or staining artifacts, especially given extensive staining trials of *Calluna vulgaris* roots using pen ink and Trypan blue stains at the individual root level. Regardless, the ErM types can easily be aggregated together by the researcher, and we do not find any drawbacks in the model performance. Recognising DSE hyphae on their own, but also when co-occurring with either ErM or AM, the trained model can better differentiate the anchor mycorrhizal classes. It also facilitates better understanding of DSE prevalence across field samples and specific habitat types, while simultaneously ensuring that the mycorrhizal classes are not underrepresented when they co-occur within a tile. There are still biological ambiguities to unpack, e.g. compressed vascular bundles mistaken for DSE hyphae, morphological variances arising from rare plants or habitats, but if the user also learns to spot these types of errors, they can easily resolve them. The contextual information added to the model by incorporating the predictions for the surrounding tiles into each tile’s classification has also boosted model predictions significantly and improved F_1_ in most classes. Negative trends were notable for the classes Type two and DSE in the ErM dataset. This was likely due to the low sample sizes for these classes, but we maintained them in the training set to support more clarity on ErM colonisation classes. As the contextual classification exploits max-voting, this could lead to other classes outnumbering the image tile with said colonisation and thus a false relabelling.

The resulting MF tool was deemed successful in its capacity to improve the pace and volume of colonisation assessments for field roots in our lab. Previously, a single root section would take half to one hour to evaluate using a microscope. Not only did this limit the number of roots per day per analyst to an average of ten, but the reproducibility under traditional microscopy methods has considerable challenges, e.g. complex root architectures make stopping and restarting in position difficult. The model can predict significantly more roots overnight (dozens to hundreds based on the hardware used). Depending on the confidence threshold designated by the user and the complexity of the root image, the expert analyser can refine these predictions to annotations in less time than traditional microscopy assessments. The tool outputs and database provide repeatability and accountability in tracking and storing the assessment scores and create a new reference visual database of roots for researchers and policy makers alike. This is a significant step towards enabling landscape-scale assessment of *in situ* mycorrhizal colonisation. However, we suggest that the resulting colonisation percentages are used as a starting point and considered within the context of experimental limitations (e.g. sampling size) and research questions. Despite the output precision, categorical assessments may be more appropriate, e.g. a DAFOR scale (Sutherland 2006) which roughly delimits Dominant (e.g. >50% of root length), Abundant, Occasional, Frequent or Rare, appropriate in real world settings. Interpreting a percent colonisation in a controlled laboratory experiment with a known mycobiont is significantly different than in roots sampled to represent change at the landscape or habitat scale. Given the link between root mycorrhizal colonisation and functional outcomes for host plants, and the responsiveness of root associated fungal communities to environmental or management change (Verbruggen et al. 2012; Soudzilovskaia et al. 2015), the tool has potential for supporting the use of mycorrhizal colonisation data as a contributing indicator of ecosystem condition and for natural capital assessments (Frew 2025). With the data we had available for training, building additional subclasses – e.g. ‘A’ (for arbuscules) and/or ‘V’ (for vesicles) vs. ‘H’ (for hyphae), would have been too complex as we then would have needed to provide class differentiation of A-only, A + V, A + H, H-only, V-only, H + V and A + H+V. We decided that differentiating DSEs and non-AM from AM presence was more useful for our research goals, i.e. identifying and quantifying AM presence at scale. Other researchers could train their images to emphasize e.g. V and A, over DSE, or *vice versa*. In any case, the model will yield summary statistics for these subcategories which may be compiled. Thus, we encourage researchers to go beyond and label functional structures i.e. vesicles and arbuscules in AM roots, which when paired with molecular data, can inform ecological responses even more. Future development of the tool can automate this feature. When coupled with an efficient low-cost staining protocol, it provides a viable option for medium-throughput colonisation assessment of field-collected roots for the first time. While some researchers did not find significant endomycorrhizal community differences in their experimental set-up between rhizophilic or edaphophilic lifestyles (Vanbruggen et al. 2012), Odriozola et al. (2026) have developed an index around predictive lifestyles to enable estimates of mycorrhizal communities based on host plants. Thus, we caution users not to draw direct inferences between rhizophilic observations and edaphophilic presence.

The requirement for expert human intervention is a double-edged sword. While it can reduce the time saving advantage of the tool, and the potential users to those capable of identifying mycorrhizal structures within roots, it allows user engagement and discovery in unravelling complexities at the cytological level, particularly useful when coupled with molecular data. We recommend a workflow which starts by setting a threshold value of 80% − 90% to allow expert time to manually focus only on tiles yielding unacceptable certainty. Future model development should focus on improving the predictive performance of the underlying machine learning model by targeting the classes that are influencing variability around estimations of AM or ErM colonisation. Since we observed a trend of improved predictive performance when adding more training data, a priority can include the labelling of additional training data especially in the underrepresented classes, e.g. ErM and DSE, and where ambiguities in morphology may occur.

Exploration of alternative model architectures such as vision transformers may enhance performance (noting that this will probably involve a trade-off with speed and computational requirements).

As there is no clear industry standard for performance-related metrics in image recognition tools, we selected a *Macro F*_*1*_ Score as the main model performance metric, because it performs well on imbalanced datasets, acknowledging multiple approaches (Opitz 2024), and represents a more holistic measure than other common metrics such as *Precision*, *Recall* or *Accuracy*. If we were to have reported test *Accuracy*, strictly defined, it would have been 91.55% for the 6-class EfficientNetB5 AM model (82.13% on the 3-class CNN, before upgrading) and 86.68% for the 10-class ErM model, but this risks overstating performance overall across classes. We therefore propose future model developments use these baseline *Macro F*_*1*_ Scores to measure performance improvement. Although *Accuracy* is a metric that can be understood intuitively, it does not provide insight into the model performance across individual classes, especially needed with the evolved MF model given its imbalanced tile class structure (Grandini et al. 2020; Opitz 2024).

Importantly, because MF is published under an open government licence, it offers strong potential for community-driven improvement. While the published version of the tool is trained on a dataset diverse enough to provide a baseline to train and evaluate most Northern European grassland or heathland systems with common AM- or ErM-hosts, researchers with a more specific study focus (by habitat or host plant, e.g. orchids) may be required to add their own image training datasets and create their own versions of the tool which are likely to be more effective for their specific system. The intuitive user interface means that this can be done without any coding or AI knowledge by uploading JPG root images and following the User Documentation to change the pixel configuration if needed. For users with programming skills, the open-source code on GitHub enables extension of both front-end and back-end functionality. This openness encourages collaborative development, accelerating model performance improvement and broadening MF’s applications, ultimately supporting high-throughput assessment of mycorrhizal colonisation across diverse ecological and practical contexts.

## Conclusions

Unsupervised, MF reaches acceptable mycorrhizal colonisation assessments across plants, fungi and habitats, especially differentiating *Uncolonised* tiles from other colonisation classes. By adding active expert human supervision, MF enables high-performing accuracy, especially with thoroughly stained intact roots. It provides users having limited prior knowledge of training machine learning models with a simple workflow to train with new image datasets and for the user to add tags to tiles further differentiating morphological or functional types within classes e.g. vesicles or arbuscules. Beyond the innovations for controlled nursery experiment root assessments, the tool’s adaptability to field-sourced variation in AM-host root biology, mycorrhizal structures and miscellaneous artifacts, and the introduction of a new model to assess habitats dominated by ericoid mycorrhizal fungi and differentiate dark septate endophytes, are of important value to researchers interested in monitoring and studying mycorrhizas in nature.

## Supplementary Information

Below is the link to the electronic supplementary material.

Supplementary file 1 (DOCX 6.40 MB)

## Data Availability

MF is publicly available under an MIT licence. Executables for Windows, MacOS and Linux are available alongside comprehensive documentation at https://doi.org/10.5281/zenodo.17979413, the images used as training and test data are available at https://doi.org/10.5281/zenodo.17476686 and the source code is available at https://github.com/MycorrhizaEcologyLab/mycorrhiza-finder/tree/v5.0.0.

## Glossary

Accuracy: Ratio of all correct predictions (true positives + true negatives) to the total number of samples. Performance models using accuracy may refer to true positives as ‘sensitivity’ and true negatives as ‘1 - specificity’.
Contextualisation: The ability of the tool to assess and incorporate the content of neighbouring regions of an image (tiles) when classifying one region.
Convolutional neural network (CNN): Machine learning architecture that is especially well-suited to extracting information from patterns in images.
Class: One of the predefined categories a CNN can assign to an input. The final layer of the CNN produces a score or probability for each class at the tile level, and the prediction outcome is the class with the highest score.
EfficientNet architecture: A family of CNNs developed specifically for making use of image resources for computer vision tasks. This process is efficient (hence the name) at capturing strong relationships with a wide receptive field at a comparatively lower number of parameters to other state-of-the-art architectures, using a compound scaling coefficient to balance network depth, width and resolution. The EfficientNet model is generally larger and more complex than the CNN1 model, providing more learning capacity for the number of parameters it uses to capture rich global features and local fine-grained features. This is especially useful for images at a size of 126x126 or 252x252 pixels, where both capturing small details and an overall understanding of the image whilst utilizing the original image resolution are important for predicting classification.
EfficientNetB5: A medium-sized member of the family of models, with around 30 million trainable parameters.
Precision: A model performance metric that represents the impact of false positives (FP), calculated at a class level as the ratio of true positive (TP) identifications of said class, to all positive identifications of the class. i.e. it quantifies the prospect of a sample actually being positive when the model classifies it as positive.
Recall: A model performance metric that represents the impact of false negatives, calculated at a class level as the ratio of true positive identifications of said class to all tiles truly of that class. i.e. it quantifies the model’s ability to spot all positive instances.
F1Score: A model performance metric that evaluates the performance of a particular class by balancing precision and recall. It is defined as the harmonic mean of precision and recall (Equation 1).
Macro F1: A model performance metric that combines the F1Scores for all classes, by taking their unweighted average, to provide insight into overall model performance (Equation 2).
Macro F1: The 3-class refers to the three classes in AMFinder *Colonised root section, Non-colonised root section, and Background*.
Max-vote system: The most probable class in the target tile is considered alongside that of four partially overlapping tiles and the target tile is assigned the class that is dominant among all five tiles.
React: A library for creating user interfaces for web applications, using the JavaScript programming language (Meta Open Source 2025).
